# Association between acetabular coverage over femoral head and rate of joint space narrowing in non-arthritic hips

**DOI:** 10.1302/2633-1462.61.BJO-2024-0143.R1

**Published:** 2025-01-15

**Authors:** Toshiyuki Kawai, Kohei Nishitani, Yaichiro Okuzu, Koji Goto, Yutaka Kuroda, Shinichi Kuriyama, Shinichiro Nakamura, Shuichi Matsuda

**Affiliations:** 1 Department of Orthopaedic Surgery, Kyoto University Graduate School of Medicine, Kyoto, Japan

**Keywords:** Hip osteoarthritis, Developmental dysplasia of hip, Femoroacetabular impingement, Joint space narrowing, Knee arthroplasty, Hip arthroplasty, Joint cartilage, joint space narrowing, arthritic hips, femoral head, hips, knee arthroplasty, Radiographs, joint space width (JSW), acetabulum, acetabular roof, lateral centre-edge angle

## Abstract

**Aims:**

This study was performed to investigate the association between the acetabular morphology and the joint space narrowing rate (JSNR) in the non-arthritic hip.

**Methods:**

We retrospectively reviewed standing whole-leg radiographs of patients who underwent knee arthroplasty from February 2012 to March 2020 at our institute. Patients with a history of hip surgery, Kellgren-Lawrence grade ≥ II hip osteoarthritis, or rheumatoid arthritis were excluded. The hip JSNR was measured, and the normalized JSNR (nJSNR) was calculated by calibrating the joint space width with the size of the femoral head in 395 patients (790 hips) with a mean age of 73.7 years (SD 8.6). The effects of the lateral centre-edge angle (CEA) and acetabular roof obliquity (ARO) in the standing and supine positions were examined using a multivariate regression model.

**Results:**

The mean JSNR and nJSNR were 0.115 mm/year (SD 0.181) and 2.451 mm/year (SD 3.956), respectively. Multivariate regressions showed that older age was associated with a larger nJSNR (p = 0.010, standardized coefficient (SC) 0.096). The quadratic curve approximation showed that the joint space narrowing was smallest when the CEA was approximately 31.9°. This optimal CEA was the same in the standing and supine positions. Multivariate regressions were separately performed for joints with a CEA of < 31.9° and > 31.9°. When the CEA was < 31.9°, a smaller CEA was associated with a larger nJSNR (p < 0.001, SC 0.282). When the CEA was > 31.9°, a larger CEA was associated with a larger nJSNR (p = 0.012, SC 0.152). The ARO was not associated with the nJSNR.

**Conclusion:**

Both insufficient coverage and over-coverage of the acetabulum over the femoral head were associated with increased joint space narrowing in hips that were non-arthritic at baseline. The effects of insufficient coverage were stronger than those of overcoverage.

Cite this article: *Bone Jt Open* 2025;6(1):93–102.

## Introduction

The incidence of hip osteoarthritis (OA) is believed to be largely related to mechanical factors.^1-3^ In particular, the coverage of the acetabulum over the femoral head is associated with the development of hip OA. Lack of sufficient coverage can lead to high joint contact force,^4^ which may subsequently result in cartilage degeneration.^5,6^ By contrast, over-coverage can cause femoroacetabular impingement (FAI).^1^ FAI is considered a risk factor for chondrolabral damage around the hip joint,^7^ which can lead to hip OA.

Although several studies have demonstrated the association between acetabular coverage and hip degeneration, most were cross-sectional in nature.^5,8-11^ Several prospective studies have demonstrated the association between acetabular morphology and the incidence of hip OA.^12-17^ A meta-analysis of prospective longitudinal evaluations showed that acetabular over-coverage was not associated with hip OA,^8^ although in some cross-sectional studies hips with OA were more likely to have a lateral centre-edge angle (CEA) of > 39°.^18,19^

In almost all of these previous studies, the outcome measurement was either the incidence of hip OA defined as Kellgren-Lawrence (KL) grade ≥ II or total hip arthroplasty.^12-17,20^ In only one study was incident radiological OA of the hip defined as a decreased width of the hip joint space (1.0 mm) at follow-up.^21^

The present study was performed to investigate the effects of acetabular coverage over the femoral head on the rate of decrease in the joint space width (JSW) over time in non-arthritic hips, and to determine the optimal degree of coverage that is associated with minimal joint space narrowing.

## Methods

This study involved a retrospective review of whole-leg standing radiographs of patients who underwent knee arthroplasty (total knee arthroplasty (TKA) or unicondylar knee arthroplasty (UKA)) at our institute (Kyoto University Graduate School of Medicine, Japan) from February 2012 to March 2020. If a patient underwent bilateral knee arthroplasty, the record for the first knee arthroplasty was included to avoid duplications. All patients provided informed consent, and the study protocol was approved by the institutional review board of our hospital. For all eligible patients, whole-leg standing radiographs including the pelvis were taken preoperatively and at follow-up.

**Fig. 1:**
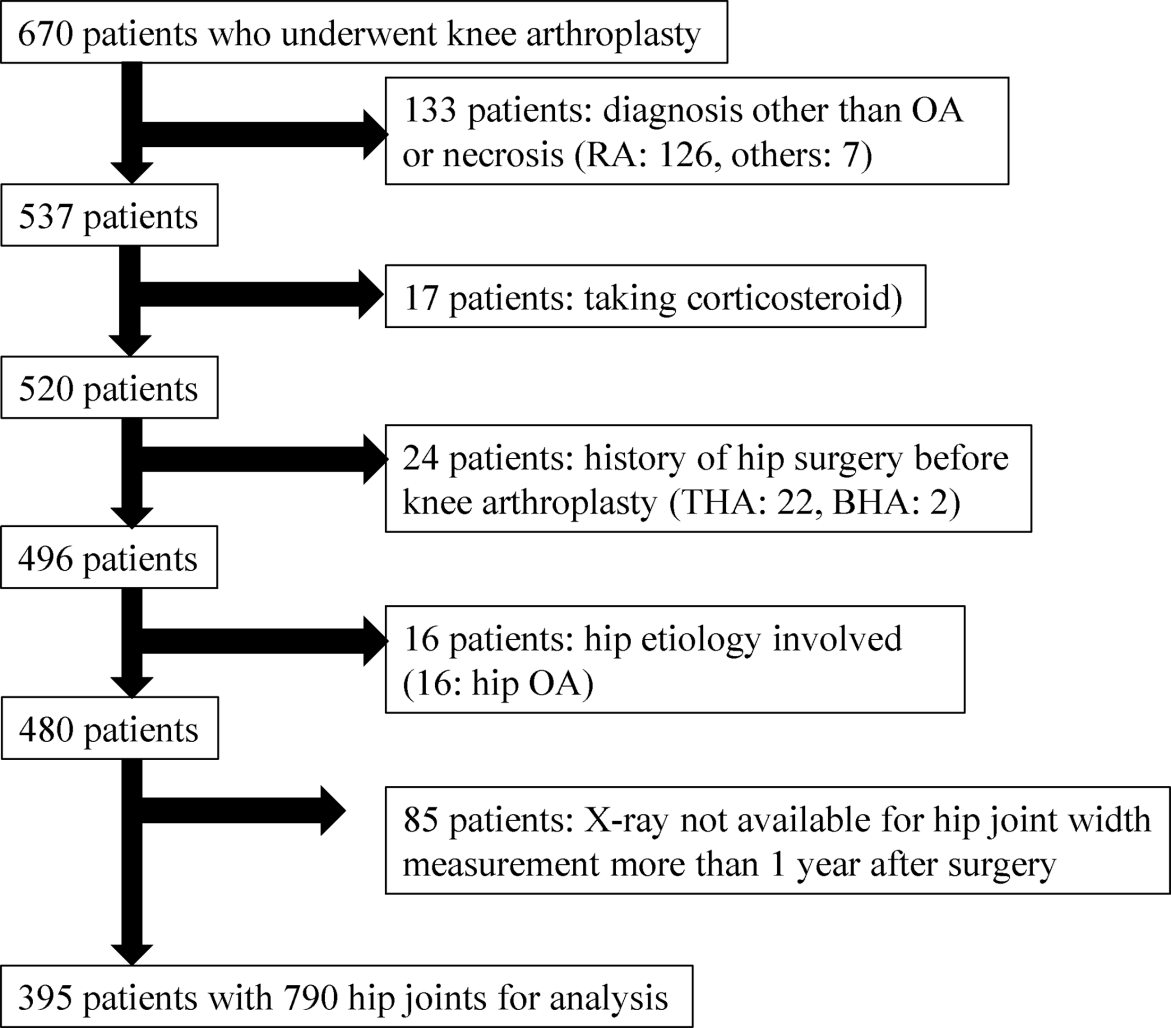
Flowchart showing inclusion and exclusion of patients. BHA, bipolar hemiarthroplasty; RA rheumatoid arthritis; OA, osteoarthritis; THA, total hip arthroplasty.

Of 670 patients who underwent TKA or UKA during the study period, 275 were excluded because they lacked available radiographs that included measurable hip joints at > one year after knee arthroplasty; had a history of hip surgery or a diagnosis other than knee OA, osteonecrosis (ON) of the femoral condyle, or hip OA (KL grade ≥ II);^22^ or lacked available postoperative whole-leg standing radiographs. The final cohort comprised 395 patients (790 hips) in whom the JSW of both hips was measurable preoperatively and at > one year postoperatively (Figure 1). The indication for knee arthroplasty was knee OA in 387 patients and ON of the femoral condyle in eight patients. In total, 345 TKAs and 50 UKAs were performed. The patients’ demographic data are shown in Table I.

### Measurements

**Fig. 2:**
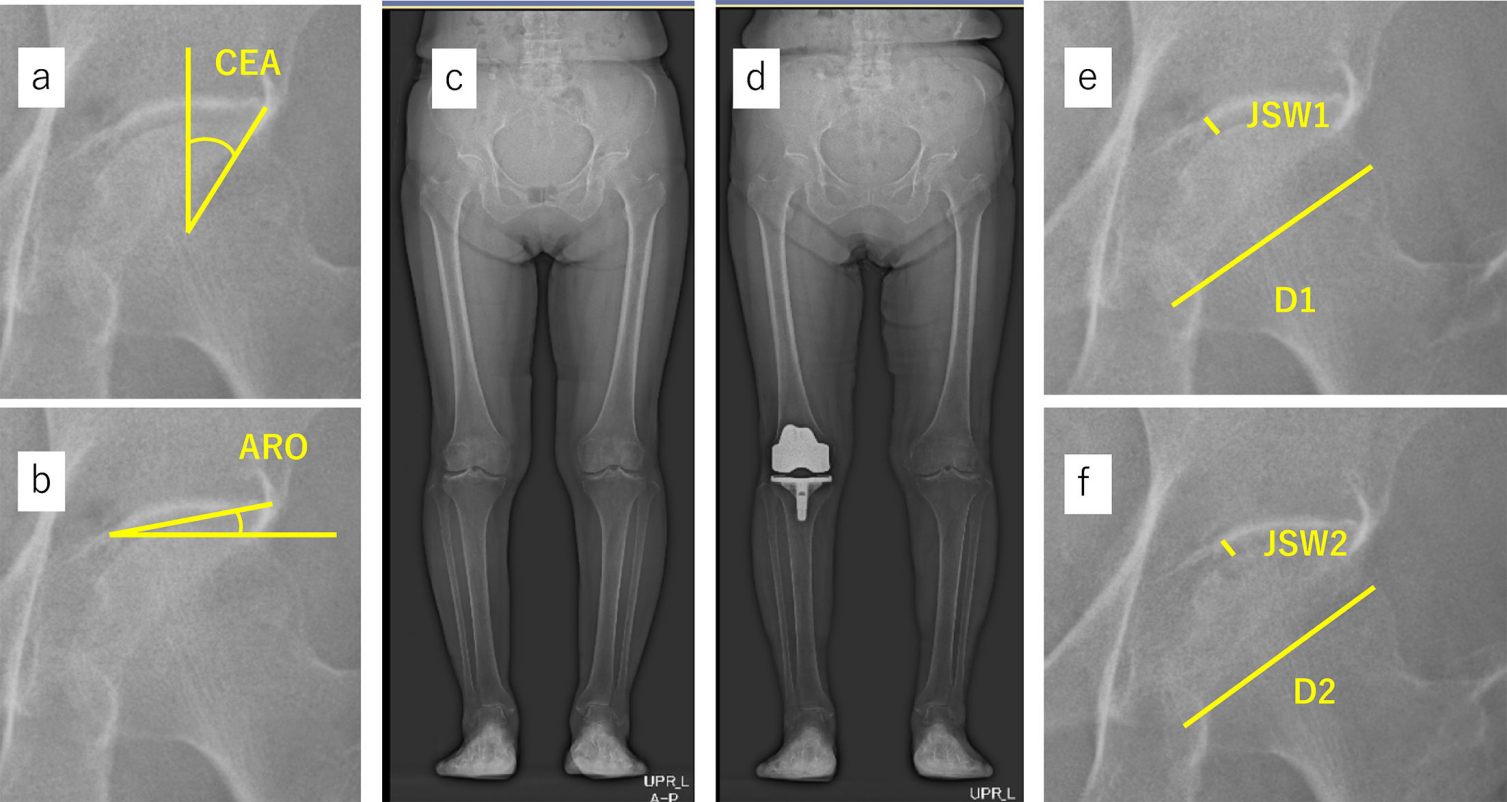
Measurements of the a) centre-edge angle (CEA), b) acetabular roof obliquity (ARO), and hip joint space width (JSW) in a representative case (75-year-old female). c) Anteroposterior (AP) standing whole-leg radiograph taken before surgery. d) AP standing whole-leg radiograph taken five years after surgery. e) Magnified image of the area including the right hip in panel c). f) Magnified image of the area including the right hip in panel d). The JSW and the femoral head diameter were measured as indicated by the yellow lines. JSW1 and JSW2 are the JSWs before surgery and at the final follow-up, respectively. D1 and D2 are the femoral head diameters measured on radiographs at three months after surgery and at the final follow-up, respectively.

The lateral CEA (Figure 2a) was defined as the angle between a line drawn from the centre of the femoral head to the lateral edge of the acetabulum and a line perpendicular to the inter-teardrop line.^23^ The acetabular roof obliquity (ARO) (Figure 2b) was defined as the angle between a line drawn from the superior point of the acetabular fossa (the end of the weightbearing region) to the lateral edge of the acetabulum.^24^ The CEA and ARO were measured on both standing and supine radiographs.

The JSW was measured on standing radiographs, as described previously.^25-27^ Briefly, the JSW was defined as the narrowest point between the cortical surface of the acetabulum and the bone contour of the femoral head on a digitized image using web-based Centricity software (GE HealthCare, USA) (Figures 2c to 2f). The joint space narrowing rate (JSNR) was defined as the decrease in the JSW between the preoperative radiograph and the latest follow-up radiograph divided by the time (in years) between the two radiographs.

To adjust for the effects of magnification of the radiograph and body size, the normalized JSNR (nJSNR) was calculated as shown below.

$$nJSNR=\frac{\left(\frac{JSW1}{D1}-\frac{JSW2}{D2}\right)}{follow-up\;duration\;in\;years}\times 1000,$$

JSW1 and JSW2 are the JSWs before knee arthroplasty and at the final follow-up, respectively, and D1 and D2 are the diameters of the femoral head measured on radiographs obtained before knee arthroplasty and at the final follow-up, respectively (Figures 2c and 2d).

The pixel size of the whole-leg radiographs was 0.23 mm. Radiographs were interpreted by an experienced orthopaedic surgeon (TK) in random order. The inter- and intrareader reliabilities were assessed in a random sample of 40 joints. The measurements for evaluation of inter-reader reliability were performed by another experienced orthopaedic surgeon (YO) who was blinded to the patients’ information.

The intraclass correlation coefficients for inter- and intrareader reliability of the femoral head size were 0.97 and 0.99, respectively, and those for the joint space measurements were 0.88 and 0.92, respectively.

### Statistical analysis

Differences in proportions were calculated using the Pearson chi-squared test. Differences in means between two groups were calculated using the Mann-Whitney U test. The Pearson correlation coefficients (*r* values) were calculated between the CEA in the standing and supine positions and between the ARO in the standing and supine positions. Univariate and multivariate regressions were performed to evaluate the associations between the hip JSNR with each of the following factors: sex, age, BMI, indication (presence of knee OA), type of arthroplasty (TKA or UKA), CEA in the standing and supine positions, and ARO in the standing and supine positions. A quadratic curve approximation was performed to detect the CEA that was associated with the smallest nJSNR, which was defined as the ‘optimal CEA’. The absolute difference between the CEA and the optimal CEA was also used as a factor, and its effect on the nJSNR was examined in the regressions. Interactions were quantified using variance inflation factors, with values of 5 to 10 indicating collinearity. Statistical significance was set at p < 0.05. All statistical analyses were performed using JMP Pro 15 software (SAS Institute, USA).

## Results

The CEA was significantly smaller in the left than right hip in the supine position (p = 0.014, Pearson correlation coefficient), whereas the difference was not significant in the standing position (p = 0.098, Pearson correlation coefficient). The ARO was larger in the right than left hip in both the supine and standing positions (p < 0.001 for both, Pearson correlation coefficient).

**Table I.** Patient demographic data.

Characteristic	Value	p-value*
Mean age, yrs (SD, range)	73.7 (8.6, 34 to 91)	
Male sex, n (%)	158 (19.0)	
Mean BMI, kg/m^2^ (SD, range)	26.0 (4.4, 13.8 to 49.3)	
Mean CEA standing total, ° (SD, range)	31.6 (6.7, 10.0 to 49.1)	
Mean CEA standing right, ° (SD, range)	32.0 (6.4, 11.6 to 49.1)	0.098
Mean CEA standing left, ° (SD, range)	31.2 (6.9, 10.0 to 52.2)	
Mean CEA supine total, ° (SD, range)	31.4 (6.8, 10.8 to 49.9)	
Mean CEA supine right, ° (SD, range)	32.0 (6.5, 11.0 to 48.4)	0.0136
Mean CEA supine left, ° (SD, range)	30.8 (6.9, 10.8 to 49.9)	
Mean ARO standing total, ° (SD, range)	6.4 (4.9, -7.4 to 23.0)	
Mean ARO standing right, ° (SD, range)	7.5 (4.7, -5.3 to 20.3)	< 0.001
Mean ARO standing left, ° (SD, range)	5.4 (4.8, -7.4 to 23.0)	
Mean ARO supine total, ° (SD, range)	6.4 (5.0, -7.5 to 21.8)	
Mean ARO supine right, ° (SD, range)	7.3 (5.1, -6.1 to 19.7)	< 0.001
Mean ARO supine left, ° (SD, range)	5.5 (4.8, -7.5 to 21.8)	
Diagnosis KOA, n (%)	387/395 (98.0)	
Type of arthroplasty (TKA/all), n (%)	345/395 (87.3)	
Mean radiograph follow-up, yrs (SD, range)	3.02 (1.80, 1.00 to 10.28)	
Mean rate of joint space narrowing, mm/yr (SD, range)	0.115 (0.181, -0.248 to 2.841)	
Mean nJSNR, mm/yr (SD, range)	2.451 (3.956, -12.775 to 57.830)	

* Mann-Whitney U test.

ARO, acetabular roof obliquity; CEA, centre-edge angle; KOA, knee osteoarthritis; nJSNR, normalized joint space narrowing rate.

The mean JSNR and nJSNR were 0.115 mm/year (SD 0.181) and 2.451 mm/year (SD 3.956), respectively.

**Fig. 3:**
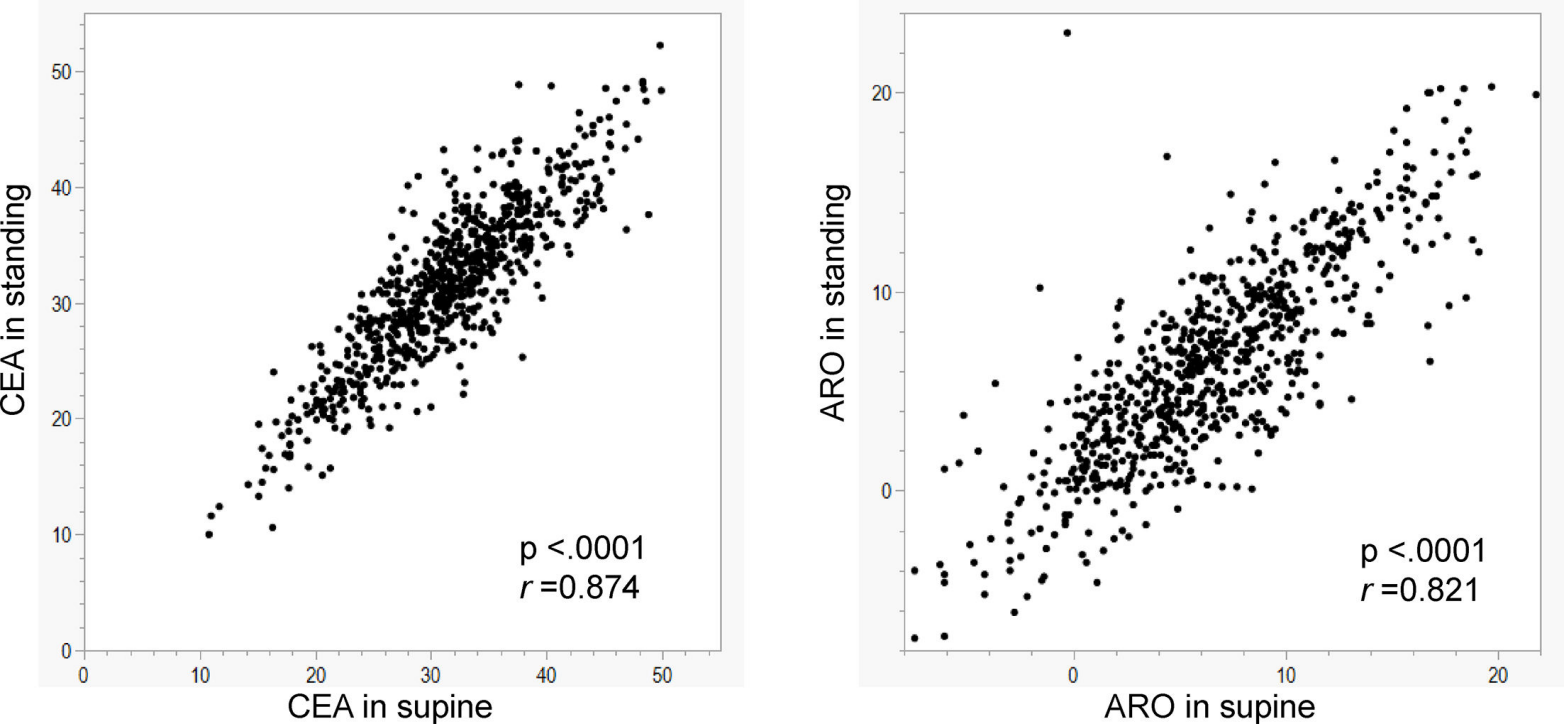
Association between hip morphology parameters in the standing and supine positions. ARO, acetabular roof obliquity; CEA, centre-edge angle.

**Fig. 4:**
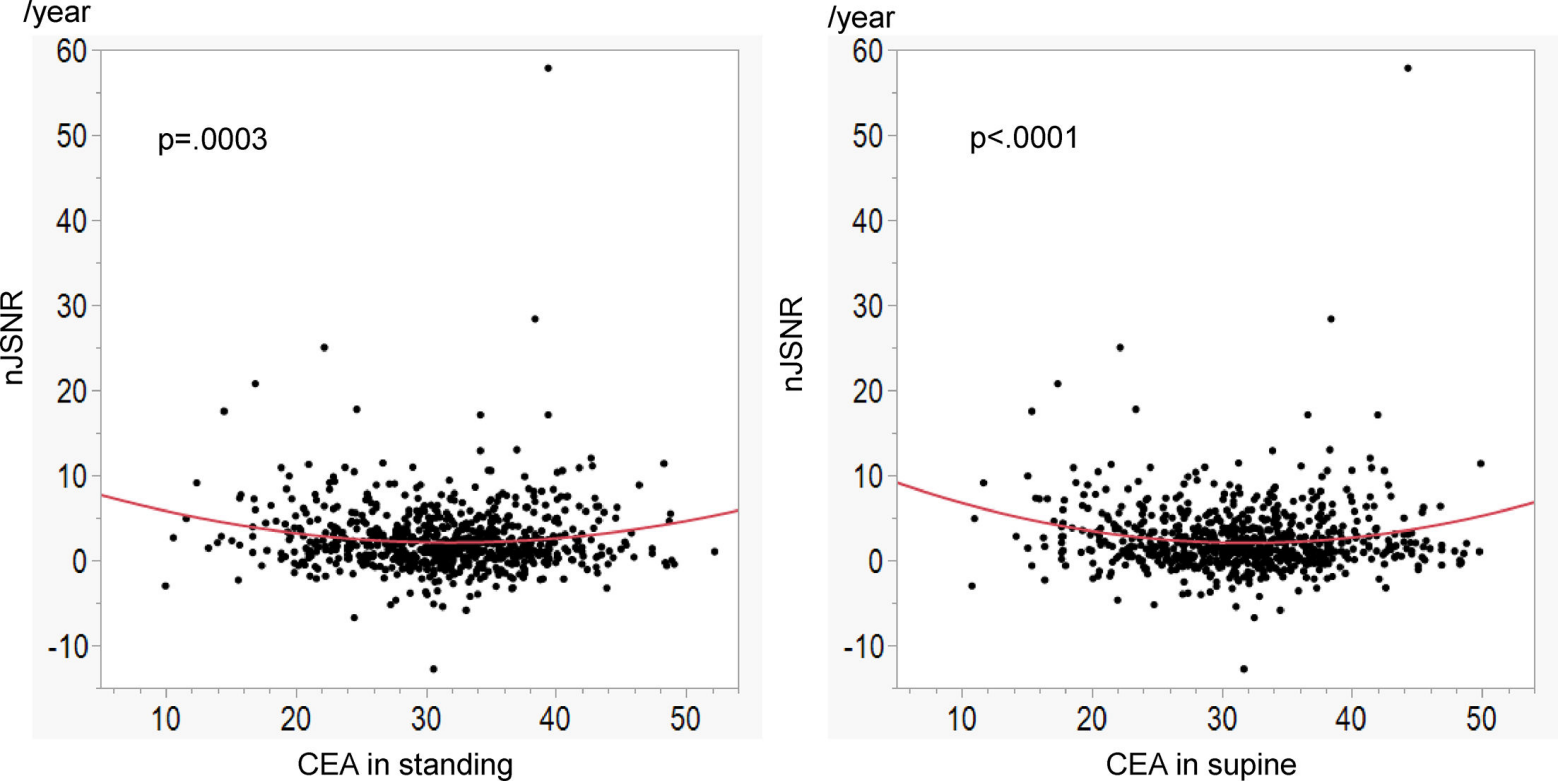
Association between the centre-edge angle (CEA) and normalized joint space narrowing rate (nJSNR). The curved lines demonstrate the quadratic curve approximation performed to evaluate the association between the CEA in each position and the nJSNR.

**Fig. 5:**
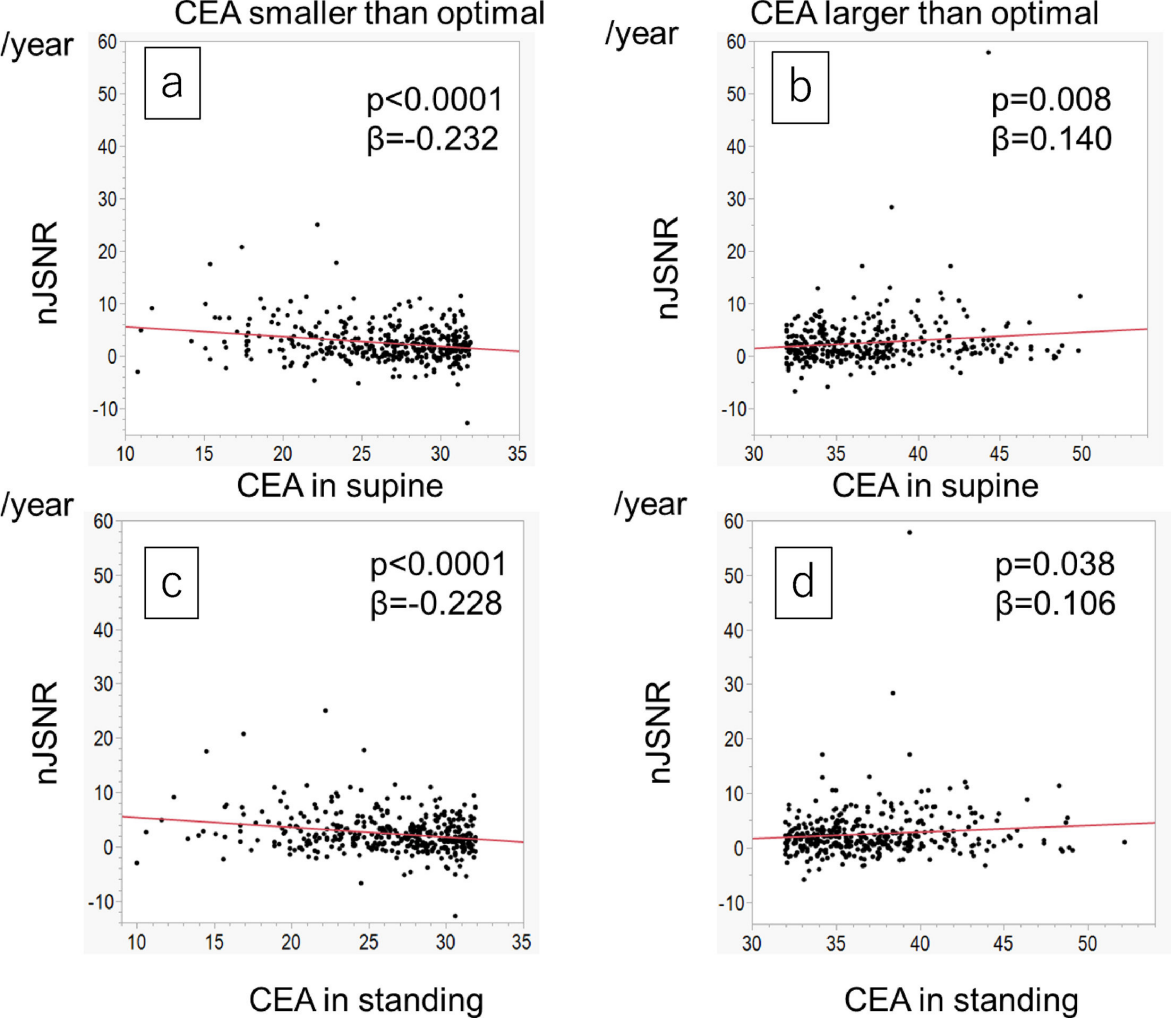
Relationship between the centre-edge angle (CEA) and normalized joint space narrowing rate (nJSNR). a) Cases with a CEA of < 31.9° in the supine position. b) Cases with a CEA of > 31.9° in the supine position. c) Cases with a CEA of < 31.9° in the standing position. d) Cases with a CEA of > 31.9° in the standing position.

The CEA in the standing position was strongly correlated with the CEA in the supine position (p < 0.0001, *r* = 0.874) (Figure 3). The quadratic curve approximation performed to evaluate the association between the CEA in the supine position and the nJSNR demonstrated that the CEA was associated with the nJSNR. The equation showed a concave upward function (Figure 4), indicating that excessive coverage and insufficient coverage were both associated with greater joint space narrowing. A CEA of 31.9° in the supine position was associated with the smallest estimated nJSNR; this CEA was defined as the optimal CEA. The CEA in the standing position that was associated with the smallest nJSNR was also 31.9° (Figure 4). The associations between other factors and the nJSNR are shown in Table II. The deviation of the CEA from the optimal CEA in both the standing and supine positions was associated with an increased nJSNR (Figure 5).

**Table II.** Univariate and multivariate regression performed with normalized joint space narrowing rate as the dependent variable.

Variable	Univariate				Multivariate				
	***t***	**Standard error**	**Standardized coefficients β**	**p-value**	***t***	**Standard error**	**Standardized coefficients β**	**p-value**	**VIF**
Sex (female)	-1.80	0.3368	-0.0608	0.072	-1.67	0.3541	-0.0606	0.096	1.033
Age	2.55	0.0203	0.0890	0.011	2.58	0.0214	0.0945	0.010	1.053
BMI (kg/m^2^)	-0.18	0.034019	-0.00632	0.858	0.18	0.0363	0.0064	0.861	1.059
Diagnosis KOA	-0.97	0.9989	-0.0341	0.330	-0.88	1.0298	-0.0323	0.380	1.058
Type of arthroplasty (TKA)	0.67	0.4253	0.0238	0.503	0.68	0.4582	0.0251	0.496	1.059
Absolute (CEA-31.9 °) supine	5.09	0.0337	0.1819	< 0.001	5.22	0.0342	0.1885	< 0.001	1.021
Absolute (CEA-31.9 °) standing	4.61	0.0335	0.1621	< 0.001					
ARO supine	-0.03	0.0290	-0.0011	0.975	-0.19	0.0295	-0.0071	0.847	1.047
ARO standing	-0.61	0.0292	-0.0218	0.541					

ARO, acetabular roof obliquity; CEA, centre-edge angle; KOA, knee osteoarthritis; TKA, total knee arthroplasty; VIF, variance inflation factor.

**Fig. 6:**
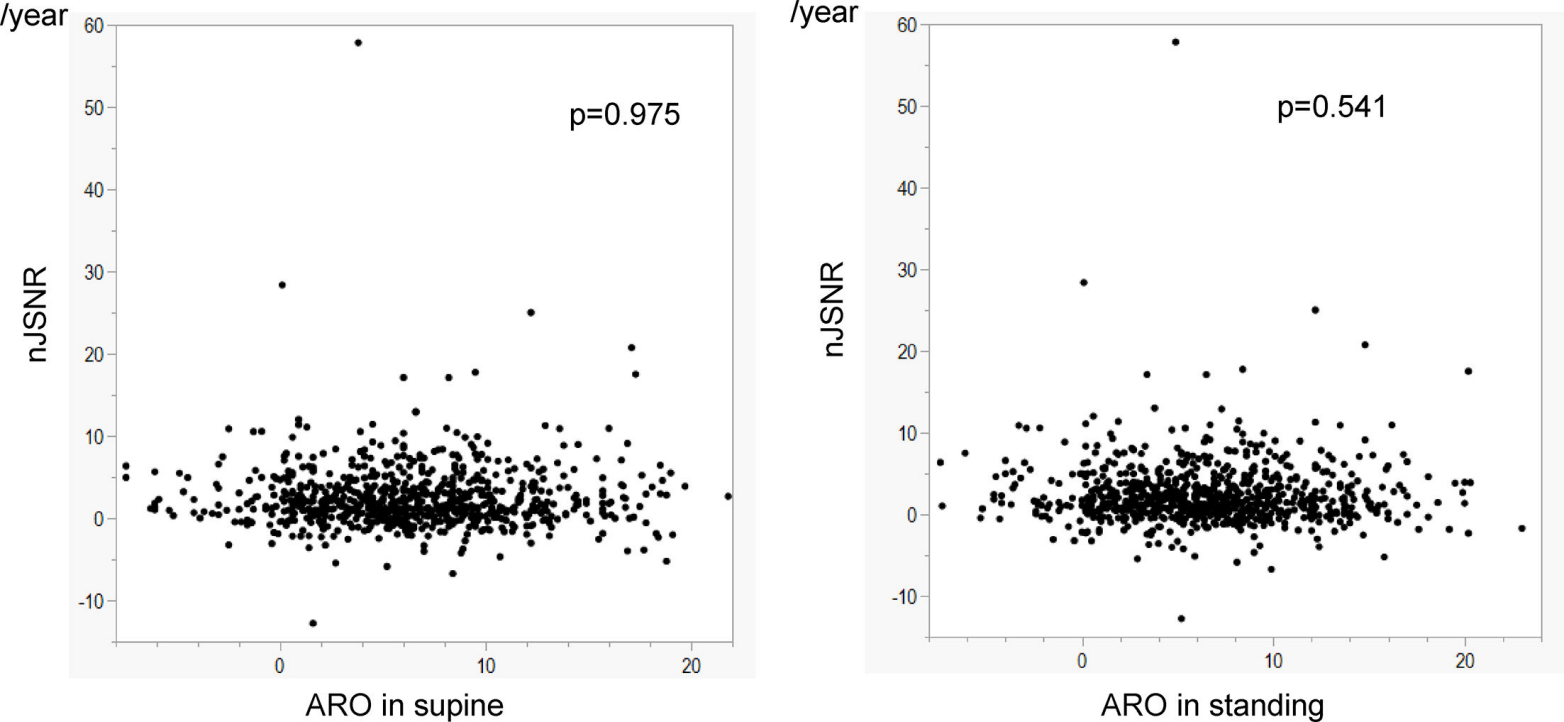
Association between acetabular roof obliquity (ARO) and normalized joint space narrowing rate (nJSNR).

The ARO in the standing position was strongly correlated with the ARO in the supine position (p < 0.001, *r* = 0.821) (Figure 3). The ARO in the standing position was not associated with the nJSNR in the linear model (p = 0.541) (Figure 6) or the quadratic curve approximation (p = 0.275). The ARO in the supine position was not associated with the nJSNR in the linear model (p = 0.975) or the quadratic curve approximation (p = 0.523).

Univariate and multivariate regression analyses were performed to evaluate patient factors, including morphological parameters (CEA and ARO), in the standing and supine positions (Table II). The CEA and ARO in the standing position were excluded from the multivariate model because of their strong correlation between the standing and supine positions (Figure 3). The absolute difference between the CEA and optimal CEA in the supine position and age were both associated with an increased nJSNR (Table II).

To examine the effects of insufficient coverage of the acetabulum over the femoral head on the nJSNR, a multivariate analysis was performed using joints with a CEA of ≤ 31.9° in the supine position (Table III). When the CEA in the supine position was < 31.9°, a smaller CEA was associated with a larger nJSNR (p < 0.001, standardized coefficient (SC) = 0.282). Male sex, an older age, and diagnosis of ON were also associated with a larger nJSNR (Table III).

**Table III.** Univariate and multivariate regression performed with normalized joint space narrowing rate as the dependent variable for joints with centre-edge angle < 31.9° in supine.

Variable	Univariate				Multivariate				
	***t***	**Standard error**	**Standardized coefficients β**	**p-value**	***t***	**Standard error**	**Standardized coefficients β**	**p-value**	**VIF**
Sex (female)	-2.05	0.4481	-0.1022	0.041	-2.25	0.4396	-0.1099	0.025	1.041
Age	2.01	0.0250	0.1005	0.045	2.04	0.0245	0.1001	0.042	1.043
BMI (kg/m^2^)	-0.94	0.0439	-0.0470	0.349	-0.76	0.0426	0.0369	0.449	1.029
Diagnosis KOA	-2.51	1.1883	-0.1247	0.013	-2.51	1.1857	-0.1247	0.013	1.073
Type of arthroplasty (TKA)	-1.02	0.5410	-0.0509	0.310	-0.19	0.5406	-0.0095	0.850	1.089
CEA supine	-4.76	0.0392	-0.2321	< 0.001	-5.03	0.0451	-0.2821	< 0.001	1.366
CEA standing	4.61	0.0335	0.1621	< 0.001					
ARO supine	1.09	0.0396	0.0546	0.276	-1.29	0.0448	-0.0734	0.197	1.400
ARO standing	1.04	0.0394	0.0520	0.300					

ARO, acetabular roof obliquity; CEA, centre-edge angle; KOA, knee osteoarthritis; TKA, total knee arthroplasty; VIF, variance inflation factor.

To examine the effects of overcoverage of the acetabulum over the femoral head on the nJSNR, a multivariate analysis was performed using joints with a CEA of > 31.9° (Table IV). When the CEA in the supine was > 31.9°, a larger CEA was associated with a larger nJSNR (p = 0.012, SC 0.152).

**Table IV.** Univariate and multivariate regression performed with normalized joint space narrowing as the dependent variable for joints with centre-edge angle > 31.9° in supine.

Variable	Univariate				Multivariate				
	***t***	**Standard error**	**Standardized coefficients β**	**p-value**	***t***	**Standard error**	**Standardized coefficients β**	**p-value**	**VIF**
Sex (female)	-0.64	0.5552	-0.0339	0.522	-0.62	0.5619	-0.0332	0.538	1.036
Age	1.43	0.0357	0.0756	0.152	1.48	0.0375	0.0819	0.140	1.101
BMI (kg/m^2^)	0.66	0.0585	0.0349	0.512	0.60	0.0618	0.0338	0.549	1.134
Diagnosis KOA	0.98	1.7271	0.0517	0.328	0.85	1.7673	0.0461	0.397	1.056
Type of arthroplasty (TKA)	1.43	0.7496	0.0750	0.155	1.02	0.7709	0.0557	0.307	1.061
CEA supine	2.68	0.0572	0.1402	0.008	2.54	0.0663	0.1521	0.012	1.288
CEA standing	2.08	0.0580	0.1058	0.038					
ARO supine	-0.95	0.0592	-0.0499	0.345	0.40	0.0677	0.0239	0.689	1.274
ARO standing	-1.71	0.0608	-0.0904	0.089					

ARO, acetabular roof obliquity; CEA, centre-edge angle; KOA, knee osteoarthritis; TKA, total knee arthroplasty; VIF, variance inflation factor.

Univariate regression analyses showed that the nJSNR was associated with age (p = 0.011) and the difference from the optimal CEA (p < 0.001) (Table II). Multivariate regression analysis also showed that older age (p = 0.010, SC 0.0945) and the difference from the optimal CEA (p < 0.001, SC 0.1885) were associated with an increased nJSNR (Table II).

**Fig. 7:**
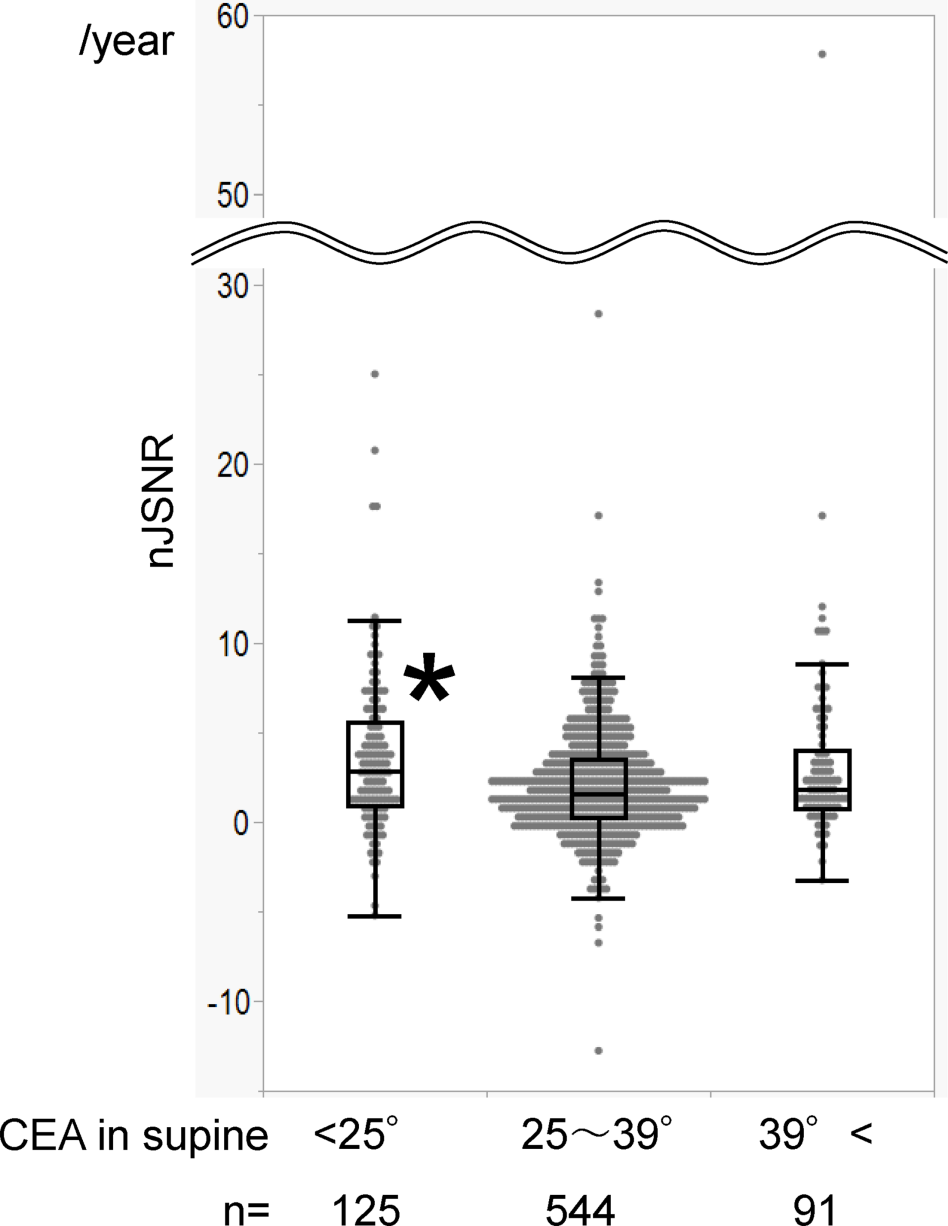
Box and whisker plots of the centre-edge angle (CEA) groups. Comparison of the normalized joint space narrowing rate (nJSNR) among the three CEA groups. The top and bottom of the boxes indicate the IQR, the line within the box indicates the median, and the whiskers represent points within 1.5 times the width of the IQR.

A separate analysis was performed for the association between CEA groups (< 25°, 25° to 39°, and > 39° in the supine position) and the nJSNR. The mean nJSNR was 3.634 mm/year (SD 4.523), 2.070 mm/year (SD 3.159), and 3.461 mm/year (SD 6.725), respectively. Univariate analysis among the three groups showed that the < 25° CEA group had a significantly larger nJSNR than the 25° to 39° CEA group (p < 0.001), whereas the difference between the > 39° group and 25° to 39° group was not significant (p = 0.170) (Figure 7).

## Discussion

In this retrospective study of patients with non-arthritic hips, the lateral CEA was associated with the hip JSNR. Our findings indicate that both insufficient coverage (CEA < 31.9°) and excessive coverage (CEA > 31.9°) are associated with increased nJSNR. Notably, the nJSNR rises as the CEA deviates from the optimal angle of 31.9° in both directions. This establishes a clear correlation between acetabular coverage and joint health, emphasizing the need for careful consideration of these parameters during surgical interventions, particularly periacetabular osteotomy.

Patients undergoing knee arthroplasty were selected for this study because the JSNR could be calculated on whole-leg radiographs, which were routinely taken before and after the knee surgery.

Two main types of hip morphology have been identified as potential risk factors for hip OA: hip dysplasia (insufficient coverage of the acetabulum relative to the femoral head) and acetabular overcoverage associated with FAI syndrome.^8^

Previous cross-sectional studies showed that a smaller CEA was associated with a smaller JSW.^9,10^ A CEA of < 25° was associated with an increased risk of conversion to total hip arthroplasty after hip arthroscopy.^28^ In the present study, when the CEA was less than the optimal CEA, a smaller CEA was associated with an increased nJSNR. Insufficient coverage can lead to high joint contact force and resultant cartilage degeneration in the hip.^4-6^ To our knowledge, no study has longitudinally examined the effect of insufficient coverage of the acetabulum over the femoral head on the hip JSNR.

In a 2022 study of Japanese volunteers, the mean CEA was 31.0° and mean ARO was 5.8°.^29^ These values are quite similar to the CEA and ARO in the present study, implying that the acetabular morphologies in our cohort reflect those in the general population of Japan.

Previous reports showed that developmental dysplasia of the hip (DDH) was more likely to occur in the left than right hip.^30-32^ In the current study, the CEA in the supine position was significantly smaller in the left than right hip; however, the difference was small (31.2° vs 32.0°).

Hoch et al^12^ reported that a CEA of < 20° was not a risk factor for hip OA in their longitudinal study. However, most other prospective studies have showed a significant relationship between DDH and hip OA. Iidaka et al^17^ reported in their prospective study that a CEA of < 20° was a risk factor for hip OA. Saberi Hosnijeh et al^20^ reported that a CEA of < 20° was a risk factor for developing hip OA in their 9.2-year follow-up. Reijman et al^21^ showed that patients with acetabular dysplasia (CEA of < 25°) had a 4.3-fold increased risk of incident radiological hip OA. Thomas et al^16^ handled the CEA as a continuous variable and reported that a smaller CEA was a risk factor for hip OA and total hip arthroplasty in their prospective study. In the present study, when the CEA was below the optimal angle of 31.9°, a smaller CEA was associated with a larger JSNR.

The Rotterdam study demonstrated that overcoverage was associated with hip OA at a follow-up of > nine years.^20^ However, a meta-analysis of three prospective studies showed no significant association between a CEA of > 39° and the development of hip OA.^8^ In the present study, when the CEA was above the optimal angle, a larger CEA was associated with a larger JSNR. The relationship was slightly weaker than when the CEA was smaller than optimal.

A cam lesion was a risk factor for hip OA in several prospective studies.^12,16,20,33^ However, neither the lateral view nor the Dunn view of the hip was available in the present cohort. Additionally, the effects of anteversion and neck-shaft angle were not examined in the present study. Previous reports showed no difference in femoral anteversion or neck-shaft angle between hips with and without OA.^8,34-36^

The ARO is a parameter often used to evaluate the acetabular morphology in patients with DDH. However, the ARO was not associated with the JSNR in the current study. The previously reported mean JSNR in non-arthritic hips was 0.114 mm/year (SD 0.168),^25^ whereas that in hips with OA ranged from 0.13 mm/year to 0.30 mm/year.^37-40^ One study showed that the JSNR in hips with established OA tended to be higher (mean 0.43 mm/year (SD 0.43)) than that in non-arthritic hips.^41^ These findings imply that the JSNR increases as OA progresses. Goker et al^42^ reported that KL radiological grade ≥ II is associated with a more rapid decline in the hip JSW. The effects of the acetabular morphology on the JSNR should be examined in hips at the same stage of OA, most ideally in non-arthritic hips. Hips with KL grade ≥ II were excluded from the present study.

In this study, higher age was associated with a larger nJSNR, although the relationship was weak (p = 0.01, SC 0.0945). Age was not associated with the JSW itself in two previous studies.^10,43^ The patients in those studies were younger (44 to 51 years) than the patients in the current study (mean 73.7 years). The effects of age could be limited even if it was statistically significant.

A previous report found no association between BMI and the hip JSNR.^25,27^ BMI was not associated with nJSNR in the current study either. Only a few reports have shown the effects of sex on the JSNR. One study showed that female sex was associated with larger JSNR, which was normalized by the size of the femoral head, than male sex,^25^ while in another study this variable was not associated with the JSNR (not adjusted by the size of the body or femoral head).^42^ In the present study, only when limited to hips with CEA less than optimal coverage, female patients had a smaller JSNR than male patients (p = 0.025, SC -0.1099).

There are several limitations to this study. First, the mean age of the cohort was 73.7 years, and patients with non-arthritic hips were included. Patients with severe dysplasia may develop OA earlier in their life and might have been excluded from this study. Second, our cohort consists exclusively of patients undergoing knee arthroplasty, which may limit the generalizability of the results to the broader population. The responses to acetabular morphology variations, particularly in relation to the CEA, may differ significantly in healthy individuals compared to those undergoing knee arthroplasty. Therefore, caution should be exercised when extrapolating these findings beyond this specific patient group. Third, the pixel size of the whole-leg radiographs was 0.23 mm. Considering that the mean joint space narrowing in this cohort was around 0.35 mm, a radiograph system with higher resolution would provide more precise measurements. A recent study has highlighted that measurements of the femoral head circumference using whole-leg radiographs are susceptible to variability depending on the imaging modality.^44^ To address this potential inconsistency in magnification ratios, our investigation primarily utilizes the ratio between femoral head size and JSW. Fourth, our study relied on 2D anteroposterior pelvis radiographs, which restricts our ability to assess 3D aspects of acetabular morphology, including acetabular version and offset. The incorporation of CT imaging could provide a more comprehensive evaluation of these parameters, enhancing the relevance of our findings in clinical practice. Fifth, while the differences in JSW were statistically significant, their clinical relevance remains uncertain. Further research is needed to determine whether these marginal differences warrant surgical interventions, such as periacetabular osteotomy, or if conservative management would be more appropriate. Finally, hip pain and function scores were not analyzed.

Both insufficient coverage and overcoverage of the acetabulum over the femoral head were associated with increased joint space narrowing in hips that were non-arthritic at baseline. The effects of insufficient coverage may be stronger than those of overcoverage.

**Take home message**

- This study investigated the link between acetabular morphology and joint space narrowing rate (JSNR) in non-arthritic hips.

- A review of radiographs from 395 patients showed that both insufficient and excessive acetabular coverage were associated with increased JSNR, with insufficient coverage having a stronger effect.

- The optimal lateral centre-edge angle for minimizing JSNR was approximately 31.9°, consistent in both standing and supine positions.

## Data Availability

The data that support the findings for this study are available to other researchers from the corresponding author upon reasonable request.
